# Comparison of cell behavior on pva/pva-gelatin electrospun nanofibers with random and aligned configuration

**DOI:** 10.1038/srep37960

**Published:** 2016-12-05

**Authors:** Chen-Yu Huang, Keng-Hsiang Hu, Zung-Hang Wei

**Affiliations:** 1 Department of Power Mechanical Engineering, National Tsing Hua University, Hsinchu City, Taiwan; 2 Institute of NanoEngineering and MicroSystems, National Tsing Hua University, Hsinchu, Taiwan

## Abstract

Electrospinning technique is able to create nanofibers with specific orientation. Poly(vinyl alcohol) (PVA) have good mechanical stability but poor cell adhesion property due to the low affinity of protein. In this paper, extracellular matrix, gelatin is incorporated into PVA solution to form electrospun PVA-gelatin nanofibers membrane. Both randomly oriented and aligned nanofibers are used to investigate the topography-induced behavior of fibroblasts. Surface morphology of the fibers is studied by optical microscopy and scanning electron microscopy (SEM) coupled with image analysis. Functional group composition in PVA or PVA-gelatin is investigated by Fourier Transform Infrared (FTIR). The morphological changes, surface coverage, viability and proliferation of fibroblasts influenced by PVA and PVA-gelatin nanofibers with randomly orientated or aligned configuration are systematically compared. Fibroblasts growing on PVA-gelatin fibers show significantly larger projected areas as compared with those cultivated on PVA fibers which p-value is smaller than 0.005. Cells on PVA-gelatin aligned fibers stretch out extensively and their intracellular stress fiber pull nucleus to deform. Results suggest that instead of the anisotropic topology within the scaffold trigger the preferential orientation of cells, the adhesion of cell membrane to gelatin have substantial influence on cellular behavior.

The behavior of cells, including cell adhesion, morphology, proliferation, and differentiation, is affected by surface topography. To create a surface with particular topography, techniques such as soft lithography^1^, photochemistry, and inkjet printing^2^ are adopted. Among them, electrospinning is a simple and versatile method of changing surface topography by creating orientation-adjustable nanofibers via adapting a different grounded platform^3^,^4^,^5^,^6^. The behavior of cells has also been found to be modulated by their microenvironments, such as soluble factors, neighboring cells, and extracellular matrix (ECM) composition for cell adhesion. Gelatin is a natural polymer similar to a kind of ECM, a derivation of collagen that is abundant in the skin, tendons, cartilage, and connective tissues of animals. Therefore, it has been extensively used for wound dressings^7^, drug delivery systems^8^, or nerves^9^. Gelatin has been widely applied due to its excellent features including high biocompatibility, biodegradability, and bioactivity^10^. The incorporation of gelatin with non-water absorptive polymers (e.g., poly(ε-caprolactone) (PCL)^11^, poly (lactic-co-glycolic acid) (PLGA)^12^, or positively charged polymers (e.g., chitosan^10^) also improves the mechanical strength of membranes for biomedical usage. Specifically, poly(vinyl alcohol) (PVA)-incorporated composites have shown strong chemical and thermal stability, and their low protein adsorption property gives them a unique adhesion property^13^,^14^. Previously, Linh *et al*. fabricated PVA-gelatin electrospinning nanofiber in water-acetic acid and deionized water solvent^15^. They studied the physical properties of nanofibers, and select aqueous solutions for dispersion to reduce the cytotoxicity of as-prepared nanofibers and improve the mechanical stability of nanofiber by gelatin. Later, Yang *et al*. evaluated the biocompatibility of PVA-gelatin nanofibers^16^. Due to the non-adhesive property but biocompatibility of PVA which limit the adhesion and spreading of cells, the comparative study of PVA and gelatin-incorporated PVA nanofibers can explore the topography-induced biological effects. Therefore, in this paper, we investigated the morphological changes of fibroblasts growing on gelatin-incorporated PVA nanofibers. Suspended aligned electrospun nanofiber with submicron thickness have been used to ignore contact effect from the supportive substrate. The results have been systematically compared the morphological changes, surface coverage, viability and proliferation of cells growing on randomly oriented or aligned nanofibers.

## Results and Discussion

**Figure 1:**
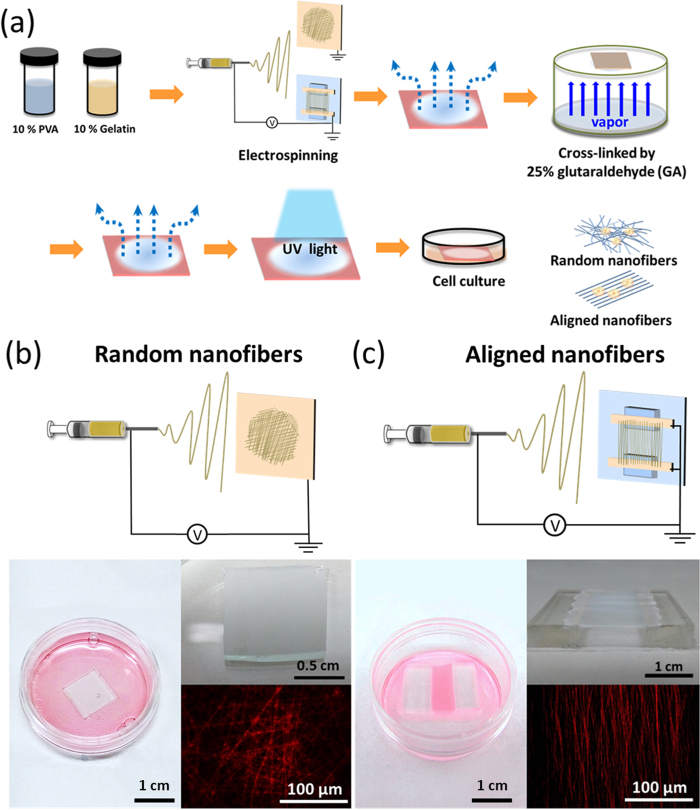
Polymer nanofibers fabricated by electrospinning on the grounded collector. (**a**) Schematics of the fabrication process of random nanofibers and aligned nanofibers. (**b**) Randomly oriented fibers were collected by the collector plate. (**c**) Aligned nanofibers were collected by parallel electrodes with a gap of 1 centimeter. Fluorescence images were taken by fibers incorporated with Rhodamine 6 G (10^−6^ M).

**Figure 2:**
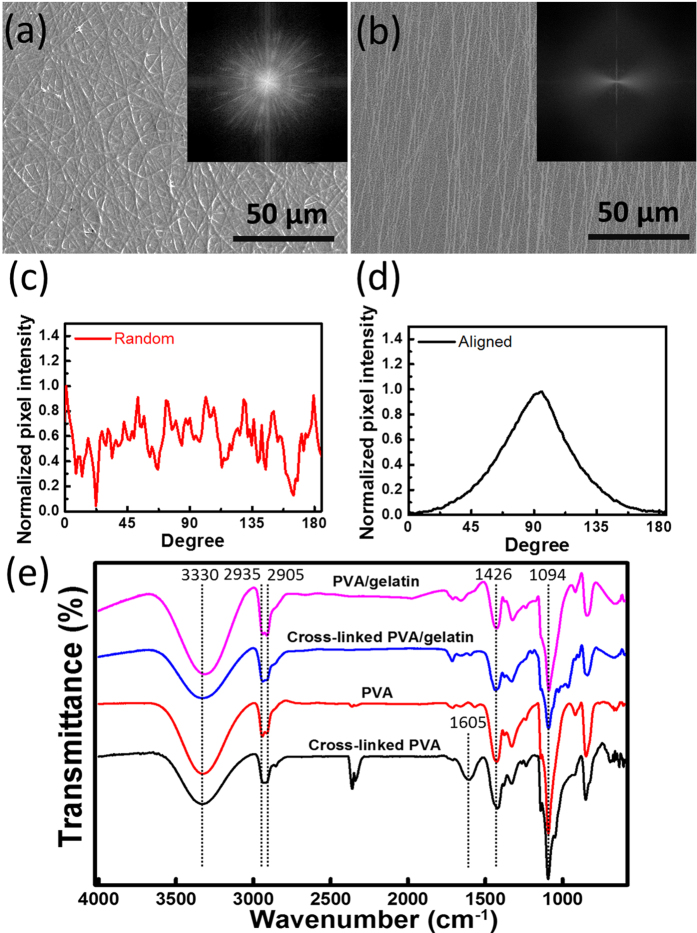
(**a**,**b**) are SEM images of random and aligned nanofibers. The insets depict frequency plots using 2D fast Fourier transform (FFT) analysis. (**c**,**d**) FFT analysis was performed on the SEM images to determine the relative degree of fiber alignment based on the conversion of the image into frequency spacing. The FFT images were analyzed with an oval-plot profile; wherein the radial intensity was summed and plotted with respect to the angle of acquisition. (**e**) FTIR spectra of random and aligned nanofibers 4000–650 cm^−1^.

Figure 1a shows the schematics of the fabrication process of random nanofibers and aligned nanofibers. The nanofibers with an average diameter of 110 nm were either randomly attached to a flat grounded collector or were aligned on parallel electrodes (Fig. 1b,c and Fig. S1). The alignment of nanofibers can be determined from the fast Fourier transform (FFT) of scanning electron microscope (SEM) images, which converts images from real space into reciprocal lattice space, as shown in Fig. 2. The FFT outputs contain grayscale patterns reflecting the degree of fiber alignment in real space, for one from aligned fibers showing a non-random and distributed pattern, while another from random fibers showing a dispersed and isotropic patterns (Fig. 2a and b). As plotted by radial intensity against the acquisition angle, the data from the aligned fibers exhibits a sharp peak, while random fibers exhibit random spikes (Fig. 2c and d).

Since PVA may be dissolved instantly in water, as-prepared nanofibers were cross-linked by glutaraldehyde vapor for 48 h soaked in a medium. Figure 2e displays the FTIR spectra of cross-linked and as-prepared PVA or PVA-gelatin nanofibers. Similar bands appear in 2935/2905 cm^−1^, representing CH_2_ asymmetric and symmetric stretching; 1605^−1^ cm is the vibration of C-O, and 1426 cm^−1^ is CH_2_ bending. The bands at 2854 cm^−1^ represent the CH stretching in the cross-linked samples, which can be attributed to the alkyl chain of aldehydes. The bands observed at 1000–1140 cm^−1^, with a broadening width after being cross-linked, are attributed to the O−C−O vibration of the acetal group. The broad bands at 3000–3600 cm^−1^, which are associated with the stretching vibration of the hydroxyl (−OH) group, indicate the existence of intermolecular and intramolecular hydrogen bonds, and the decreased band intensity after the cross-linking process shows the formation of the −OH group’s acetal bridge.

**Figure 3:**
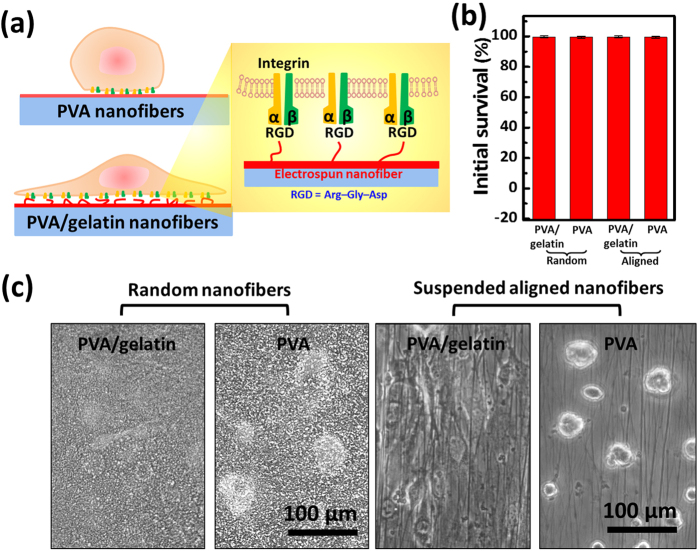
PVA and PVA-gelatin nanofibers and cell growth. (**a**) Schematics of cell attachment on PVA and PVA-gelatin nanofibers (**b**) Initial survival of cells after seeding on the nanofibers for 24 h. (**c**) cell morphology of growing cell on the PVA and PVA-gelatin random and aligned fibers.

Following the initial characterization of the electrospun fibers, we then focused on cellular interactions with the electrospun fibers. In order to ignore the contact effects from the underneath glass substrate, suspended aligned electrospun nanofiber anchored to two separates PDMS have been used to culture cells. The interaction between the cell membrane receptors and ligands of the contact fiber matrix is often the initial step in the formation of cell-matrix adhesion. The 3T3 fibroblasts can modulate the local ECM to promote their adhesion and migration and to regulate cellular functions. However, due to the low protein affinities of PVA which limit the binding of secreted ECM to support cell attachment, cells appear rounded as grown on PVA fibers (Fig. 3). In contrast, due to the arginine-glycine-aspartic acid (RGD) integrin-binding sequence of gelatin, including the Aα-chain and the heparin binding domain within the Bβ-chain which mediates cell–matrix interactions (Fig. 3a), the 3T3 cells can adhere and stretch out after being cultured on PVA-gelatin fibers (Fig. 3c). However, even though the cells have distinctive adhesive properties on PVA and PVA-gelatin fibers, they all remain highly viable after the seeding on the scaffold in the initial 24 h (Fig. 3b). The results indicate that both PVA and PVA-gelatin scaffolds does not appear significant toxicity.

**Figure 4:**
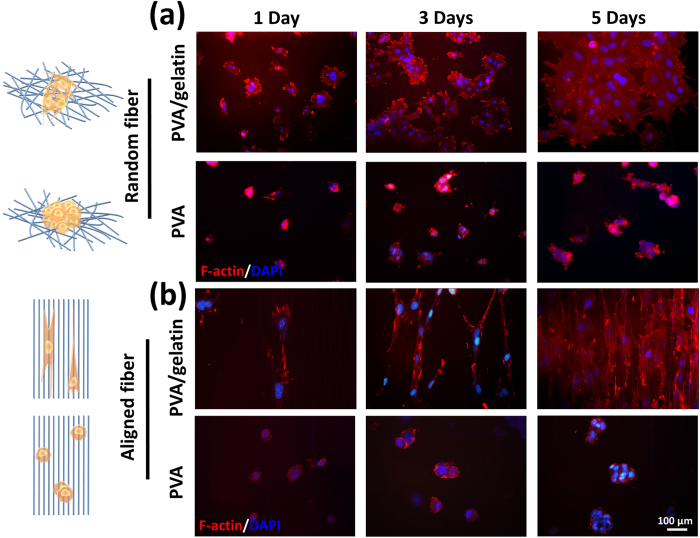
Fluorescence merged image of 3T3 fibroblast cells cultivated on random and aligned PVA and PVA-gelatin meshes for 1, 3, and 5 days. The respective image of fibroblast cells grew on (**a**) random and (**b**) aligned fibers. Fluorescence images visualize detailed cytoskeleton morphology of fibroblast cells. F-Actin stained by anti-F-actin-cy3 (red) and nuclei stained with DAPI (blue).

To learn whether the orientation of underlying nanofibers affected the cell adhesion, 3T3 cells were fixed and stained to visualize the patterns of filamentous actin (F-actin) after culture for 1, 3, and 5 days. The morphologies are distinct for 3T3 cells growing on PVA nanofibers and on PVA-gelatin nanofibers, as seen in Fig. 4. Cells growing on both random and aligned PVA nanofibers show unstretched shorter F-actin stress fiber bundles while attached and stretched out extensively on PVA-gelatin nanofibers. Cells also formed confluent layers on both aligned and random PVA-gelatin nanofibers after long-term culture, while they formed a closely compact structure with restricted colonies on PVA nanofibers.

**Figure 5:**
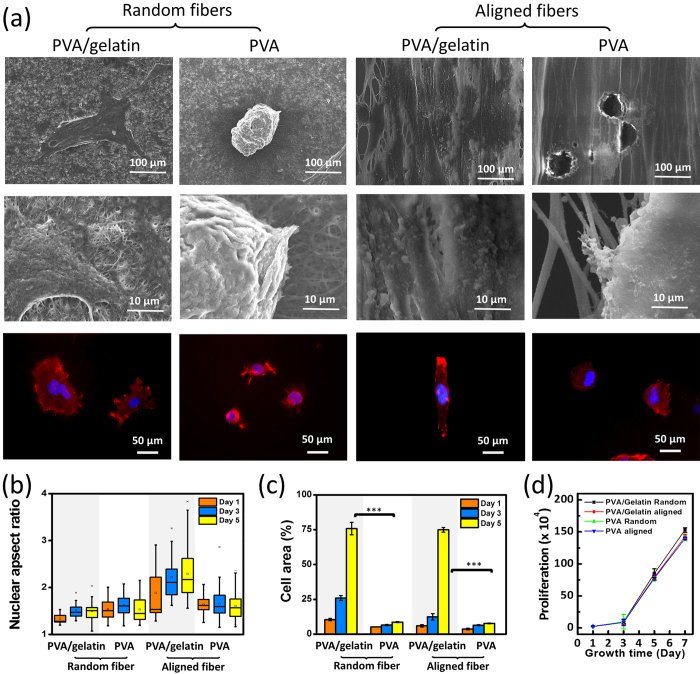
(**a**) Scanning microscope images and fluorescence merged image of 3T3 fibroblast cells cultivated on random and aligned PVA and PVA-gelatin meshes. Fluorescence images visualize detailed cytoskeleton morphology of fibroblast cells. Actin stained by anti-F-actin-cy3 (red) and nuclei stained with DAPI (blue). (**b**) Nuclear aspect ratio and (**c**) projected area of the cells growing on random and aligned PVA and PVA-gelatin meshes for 1 to 5 days. ***Indicate statistically significant differences with P value < 0.005. (**d**) Cell proliferation.

The microstructure of cells observed by SEM gives more information about cell-matrix contacts (Fig. 5a). Those cells growing on PVA-gelatin nanofibers exhibit more isotropic morphologies on random fibers but show as elongated along the fibers with flattened morphology, and less clear borders appear between the cells and nanofiber. Conversely, cells show minimum interaction with surrounding matrices as cultured on PVA fibers. The spatial structures of the F-actin fibers can be compared with fluorescent images under larger magnification. The F-actin fibers of cells growing on random PVA-gelatin fibers were rather stochastically distributed, while the F-actin fibers of cells growing on aligned PVA-gelatin fibers assembled in alignment with the direction of the nanofibers, resulting in a uniform linear pattern coinciding with the polarized cell growth. From side view of growing cells on suspended PVA-gelatin nanofibers (Supplementary Fig. S2), the cell membrane fuse nanofibers and suggest the membrane integrin diffuse to adhere to the underlying fiber and limit the lateral expansion of cell bodies.

In Fig. 5b, the increase of nuclear aspect ratio with the culture time for cells growing on aligned PVA-gelatin fibers reveal the impact of topography of nanofibers on cell growth. The stretching of the cell is coupled with nuclear deformation caused by the lateral pulling stress from the F-actin fiber transmitted to the cell nucleus, and therefore, the nuclear aspect ratios of cells growing on PVA-gelatin aligned fibers are found to be higher than others. The difference in the spreading among cells growing on different nanofibers can be further represented by their projected areas. As illustrated in Fig. 5c, cells growing on PVA-gelatin fibers show significantly larger projected areas as compared with those cultivated on PVA fibers, whose p-value was smaller than 0.005. As shown in in Fig. 5d, the cells on PVA fibers with random or align configuration can still retain the ability to grow and divide, and only slightly decreased in the number after growing for 7 days as compared with the group without gelatin coating. From the results, the low protein affinities of PVA does not show strong negative influences on cells proliferation.

In summary, the binding of cells via integrin to the RGD sequence of gelatin have substantial influences on cell behavior. Previous results have shown that 3T3 fibroblasts can grow along preferable directions on optic media with microgroove patterns without ECM coating^17^. Such substrates give more homogeneous surface stress at both sides and bottom of cell membrane and served as physical barrier to limit the lateral expansion of cell bodies and alter the preferential orientation of cells. However, from the results of aligned PVA and PVA/gelatin nanofibers, the adhesion of cell membrane to suspended nanofibers via gelatin is vital to transduce physical external stimuli to cells and have substantial influence on cellular behavior. Moreover, though cells attach across several subcellular scale nanofibers would not experience homogeneous surface stress, the underlying gelatin nanofibers give repetitive guidance to direct membrane integrin diffusion along one direction and limit the lateral expansion of cell bodies^18^. In contrast, the cells grow on PVA electrospun fiber lack of extra binding protein but rely mainly on the modulation of secreted ECM to promote cell adhesion. The low protein affinities of PVA limit the absorption of ECM to support cell attachment, cells appear rounded as grown on PVA fibers and gradually form multicellular compact structures.

## Conclusion

The comparative study of randomly oriented or aligned PVA and PVA-gelatin nanofibers and their topography-induced biological effects have been explored. Fibroblasts were able to form confluent layers on both aligned and random PVA-gelatin nanofibers, while they formed a closely compact structure with restricted colonies on PVA nanofibers. Cells on PVA gelatin-aligned fibers stretched out extensively, and their intracellular stressed fibers pulled the nucleus to deform. The initial survival and the long-term cultivation suggested that the low protein affinities of PVA limit the absorption of ECM to support cell attachment but does not show strong negative influences on cells proliferation. Instead of the anisotropic topology within the scaffold trigger the preferential orientation of cells, the adhesion of cell membrane to gelatin have substantial influence on cellular behavior.

## Materials and Methods

### Fabrication of random and aligned PVA nanofiber

Initially, 10% w/v of poly(vinyl alcohol) (PVA) (Mw: 70,000–80,000, First Chemistry) and 10% w/v of gelatin (G1890, Sigma) was prepare in deionized water. HCl solution was added to create base PVA. Base PVA was then mixed with gelatin solution (80/20 w- w) stirred at 120 °C for 1 h and then cooled to room temperature with continuous stirring overnight. The fibers were spun at 15 kV with a feeding rate of 0.005 mL/min, together with a blunt-ended 21 gauge needle as the spinneret at a distance of 15 cm. For random fiber, a piece of copper plate was used as a collector. Aligned nanofiber scaffolds were fabricated utilizing a collector consisting of a parallel plate electrodes were 1 cm apart and attach to 2 cm × 1 cm × 0.5 cm (l × w × h) polydimethylsiloxane (PDMS). Then, the electrospun nanofibers were dried at 120 °C for 2 h. Since PVA would be dissolved instantly in water, the nanofibers were cross-linked by glutaraldehyde vapor (25% aq. soln., Alfa Aesar) for 24 h and followed by dried at 120 °C for 12 h to reduce residual glutaraldehyde. Subsequently, the samples were sterilized by UV exposure 30 mins and were rinsed with PBS for 24 h before cell seeding.

### Cell cultures and cell seeding on nanofibers

3T3 mouse fibroblast (BCRC No. 60071) were cultured in 90% Dulbecco’s modified Eagle’s medium (DMEM) with 4 mM L-glutamine, 1.5 g/L sodium bicarbonate, 4.5 g/L glucose, 10% bovine calf serum (BCS), and (100 U/mL penicillin and 100 μg/mL streptomycin) (Bioscience). The cell line was maintained at 37 °C in 5% CO_2_ in a humidified atmosphere and passaged to keep the cell in exponential growth. Cells were trypsinized, and adjusted to a concentration of 2 × 10^5^/mL. 100 μL of cell suspension was dropped to fully cover the surface of 1 cm^2^ nanofiber scaffolds and incubated at 37 °C in 5% CO_2_ for 3 h and supply the wells with additional 1 mL medium.

### Cell viability

To determine the cell viability on the scaffold in the initial 24 h, the 1 cm^2^ nanofiber scaffolds were incubated with 0.05% trypsin–0.01 EDTA solution for 5 mins at 37 °C to detach cells. Following by neutralized with DMEM and centrifuged to replace the medium with fresh DMEM, cell viabilities were accessed by trypan blue exclusion.

### Cell proliferation

MTT assay was performed to analyze cell proliferation of cells growing on 1 cm^2^ nanofiber scaffolds at day 1, day 3, day 5, and day 7. Serial dilutions of cells (10000, 20000, 30000, 50000, 125000, 200000, 225000 cells/100 μL) were seeded at 37 °C for 12 hours for MTT standard curve. The reagent of MTT, 3-[4,5-dimethylthiazol-2-yl]-2,5-diphenyl-tetrazolium bromide (Alfa Aesar) was dissolved in PBS at a concentration of 5 mg/mL as a stock solution. Later, MTT assay was performed to establish the correlation between cell number and signal produced. Later, the culture medium was substituted by DMEM contain 10% v/v of MTT reagent and were incubated back into the incubator for an additional 4 h. After that, the medium was changed to 100% Dimethyl sulfoxide (DMSO) solution to dissolve the insoluble purple formazan product, and the absorbance at 570 nm was recorded by a microplate reader. The standard curve was then plotted and fitted with linear regression to evaluate the cell proliferation (Supplementary Fig. S3).

### Scanning electron microscopy

Nanofibers were deposited with 3 nm gold thin film via e-beam evaporation, and the morphology of the surfaces were analyzed by JSM-6390 scanning electron microscope. SEM images were converted to 8-bit grayscale and were processed with ImageJ software supported by an oval profile plug-in (http://rsb.info.nih.gov/ij/plugins/oval-profile.html)^19^. The FFT images were then analyzed with an oval profile plug-in, in which the radial intensity was summed and plotted against the acquisition angle and data was normalized to a baseline value of 0, allowing different data sets to be directly compared^20^. Scaffolds seeded with cells were followed by conventional fixation process, in short, they were firstly rinsed with phosphate buffered saline (PBS) twice and followed by covered with 2.5% Glutaraldehyde (GA) solution for 30 minutes. Subsequently, scaffolds were rinsed with PBS, dehydrated with ethanol gradient (40, 50, 70, 95, and 100%).

### Attenuated total reflectance-Fourier transform infrared (ATR-FTIR) spectroscopy

The spectra of random/aligned nanofibers were obtained to compare the sample before and after crosslinked by glutaraldehyde. FTIR spectrometer (Vertex 80 v; Bruker Optics) with horizontal attenuated total reflectance (ATR) ZnSe cell and the DTGS-KBR detector were used. Nanofibers were placed onto the crystal cell and then clamped into the mount of the FTIR spectrometer. The spectral range of 600–4000 cm^−1^ was recorded by automatic gain signals from 200 scans with a resolution 1 cm^−1^. The data was normalized to the background spectrum recorded from a clean empty cell.

### Immunofluorescence staining

Cells were fixed with 3.7% paraformaldehyde (PFA) for 10 minutes, washed in PBS (phosphate buffered saline), permeabilized for 30 sec with cold Acetone/Ethanol at −20 °C and then blocked with 1% BSA (bovine serum albumin) for 30 minutes. The cells were washed with PBS and subsequently incubated with primary F-actin antibodies (Sangon) (1:200 v:v dilutions in 1% BSA) for 1 h at 37 °C, and followed by anti-Rabbit-cy3 secondary (Sangon) antibody (1:300 v:v dilution in 1% BSA solution) for 1 h at 37 °C, DAPI (1:1000 dilute) to stain for F-actin and nuclei. Cells were imaged with an inverted fluorescence microscope (Olympus CKX41) which was equipped with blue, green & UV filters.

### Morphological and Statistical Analysis

Nuclear aspect ratio was analyzed by taking 20 DAPI staining fluorescence images under magnification of 200 X. Image J 14.45 (NHI, Bethesda, MD, USA; http://rsbweb.nih.gov/ij/) with NMA plug-in (http://www.ufrgs.br/labsinal/nma/) was used^21^. Projected area was calculated by the F-actin staining fluorescence images with the region of interest (ROI) manager. All data were expressed as mean ± standard deviation (SD). Statistical significance of differences between the means was determined using Student’s t-test.

## Additional Information

**How to cite this article**: Huang, C.-Y. *et al*. Comparison of cell behavior on pva/pva-gelatin electrospun nanofibers with random and aligned configuration. *Sci. Rep.* **6**, 37960; doi: 10.1038/srep37960 (2016).

**Publisher's note:** Springer Nature remains neutral with regard to jurisdictional claims in published maps and institutional affiliations.

## Supplementary Material

Supplementary Information

## References

[b1] Qin D., Xia Y. & Whitesides G. M. Soft lithography for micro- and nanoscale patterning. Nat. Protoc. 5, 491–502 (2010). 20203666 10.1038/nprot.2009.234

[b2] Sheets K., Wunsch S., Ng C. & Nain A. S. Shape-dependent cell migration and focal adhesion organization on suspended and aligned nanofiber scaffolds. Acta Biomater. 9, 7169–7177 (2013). 23567946 10.1016/j.actbio.2013.03.042

[b3] Dersch R., Liu T., Schaper A. K., Greiner A. & Wendorff J. H. Electrospun nanofibers: Internal structures and intrinsic orientation. J. Polym. Sci. A Polym. Chem. 41, 545–553 (2003).

[b4] Zussman E., Theron A. & Yarin A. L. Formation of nanofiber crossbars in electrospinning. Appl. Phys. Lett. 82, 973–975 (2003).

[b5] Sundaray B., Subramanian V. & Natarajan T. S. Electrospinning of continuous aligned polymer fibers. Appl. Phys. Lett. 84, 1222–1224 (2004).

[b6] Katta P., Alessandro M., Ramsier R. D. & Chase G. G. Continuous Electrospinning of Aligned Polymer Nanofibers onto a Wire Drum Collector. Nano Letters 4, 2215–2218 (2004).

[b7] Choi Y. S. . Study on gelatin-containing artificial skin: I. Preparation and characteristics of novel gelatin-alginate sponge. Biomaterials 20, 409–417 (1999). 10204983 10.1016/s0142-9612(98)00180-x

[b8] Akin H. & Hasirci N. Preparation and Characterization of Crosslinked Gelatin Microspheres. J. Appl. Polym. Sci. 58, 95–100 (1995).

[b9] Li X. K. . Characteristics of PLGA-gelatin complex as potential artificial nerve scaffold. Colloids Surf. B 57, 198–203 (2007). 10.1016/j.colsurfb.2007.02.010 17368867

[b10] Wan Y., Wang Y., Cheng G. & Yao K. Preparation and characterization of gelatin gel with a gradient structure. Polym. Int. 49, 1600–1603 (2000).

[b11] Chong E. J. . Evaluation of electrospun PCL/gelatin nanofibrous scaffold for wound healing and layered dermal reconstitution. Acta Biomater. 3, 321–330 (2007). 17321811 10.1016/j.actbio.2007.01.002

[b12] Nagahama H. . Preparation and characterization of novel chitosan/gelatin membranes using chitosan hydrogel. Carbohydr Polym. 6, 255–260 (2009).

[b13] Weis C. . Poly(vinyl alcohol) membranes for adhesion prevention. J. Biomed. Mater. Res. B Appl. Biomater. 70, 191–202 (2004). 15264300 10.1002/jbm.b.30007

[b14] Pelipenko J. The topography of electrospun nanofibers and its impact on the growth and mobility of keratinocytes. Eur. J. Pharm. Biopharm. 84, 401–411 (2013). 23085581 10.1016/j.ejpb.2012.09.009

[b15] Linh N. T. B., Min Y. K., Song H.-Y. & Lee B. T. Fabrication of polyvinyl alcohol/gelatin nanofiber composites and evaluation of their material properties. J. Biomed. Mater. Res. 95B, 184–191 (2010). 10.1002/jbm.b.31701 20737434

[b16] Yang C., Wu X., Zhao Y., Xu L. & Wei S. Nanofibrous scaffold prepared by electrospinning of poly(vinyl alcohol)/gelatin aqueous solutions. J. Appl. Polym. Sci. 121, 3047–3055 (2011).

[b17] Anene-Nzelu C. G. . Scalable cell alignment on optical media substrates. Biomaterials 34, 5078–87 (2013). 23601659 10.1016/j.biomaterials.2013.03.070

[b18] Dang J. M. & Leong K. W. Myogenic Induction of Aligned Mesenchymal Stem Cell Sheets by Culture on Thermally Responsive Electrospun Nanofibers. Adv. Mater. 19, 2775–2779 (2007). 18584057 10.1002/adma.200602159 PMC2440686

[b19] O’Connell B. Oval Profile Plot. (Date of access: 1/30/2014) (https://imagej.nih.gov/ij/plugins/oval-profile.html) (2002).

[b20] Ayres C. Modulation of anisotropy in electrospun tissue-engineering scaffolds: Analysis of fiber alignment by the fast Fourier transform. Biomaterials 27, 5524–5534 (2006). 16859744 10.1016/j.biomaterials.2006.06.014 PMC2929953

[b21] Filippi-Chiela E. C. . Nuclear Morphometric Analysis (NMA): Screening of Senescence, Apoptosis and Nuclear Irregularities. PLoS One 7, e42522 (2012). 22905142 10.1371/journal.pone.0042522 PMC3414464

