# Supplementary material for: Comparison of cell behavior on pva/pva-gelatin electrospun nanofibers with random and aligned configuration
Source: Sci Rep. 2016 Dec 5;6:37960. doi: 10.1038/srep37960 (PMC5137148; doi:10.1038/srep37960)
Supplement: Supplementary Information [file srep37960-s1.doc]

**Supplementary**

**Title: Comparison of cell behavior on pva/pva-gelatin electrospun nanofibers with random and aligned configuration**

Chen-Yu Huang^1^, Keng-Hsiang Hu^2^ and Zung-Hang Wei^1,*^

*^1^Department of Power Mechanical Engineering, National Tsing Hua University, Hsinchu City, Taiwan.*

*^2^Institute of NanoEngineering and MicroSystems, National Tsing Hua University, Hsinchu, Taiwan.*

* [wei@pme.nthu.edu.tw](mailto:wei@pme.nthu.edu.tw)


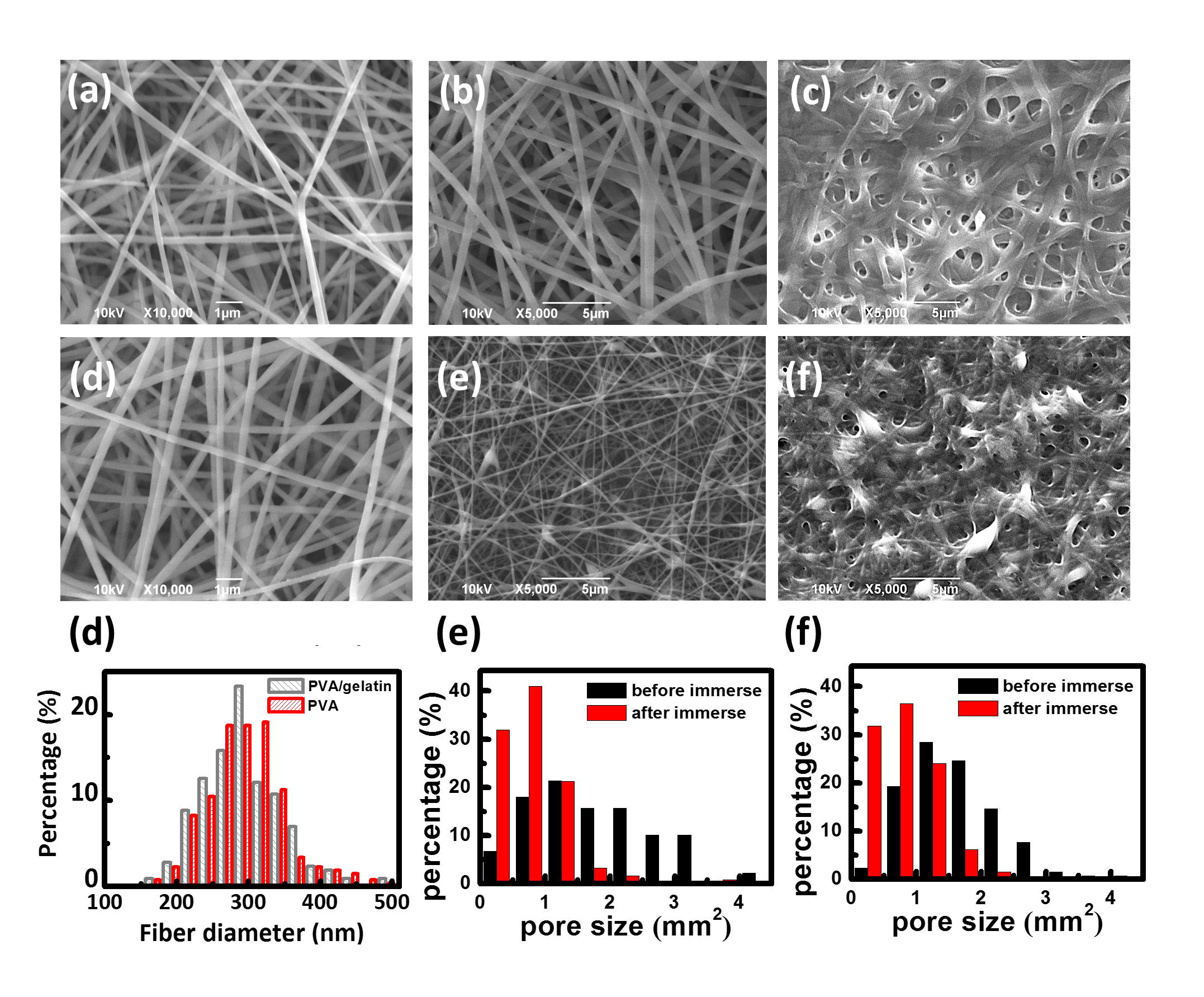


**Figure S1**. Morphologies of PVA fibers and PVA-gelatin fibers (a) (d) as-prepared PVA fibers and PVA-gelatin (b)(e)after cross-linked by glutaldehyde (c)(f) after soaked in medium for a week (g) fiber diameter of as-prepared PVA and PVA-gelatin fibers (h) pore size of mesh. After soaked in medium, the hole of crosslinked PVA will be reduced from 1.76 ± 0.94 mm^2^ to 0.76 ± 0.49 mm^2^, and that for PVA-gelatin meshes will be reduced from 1.61 ± 0.71 to 0.79 ± 0.45 mm^2^.


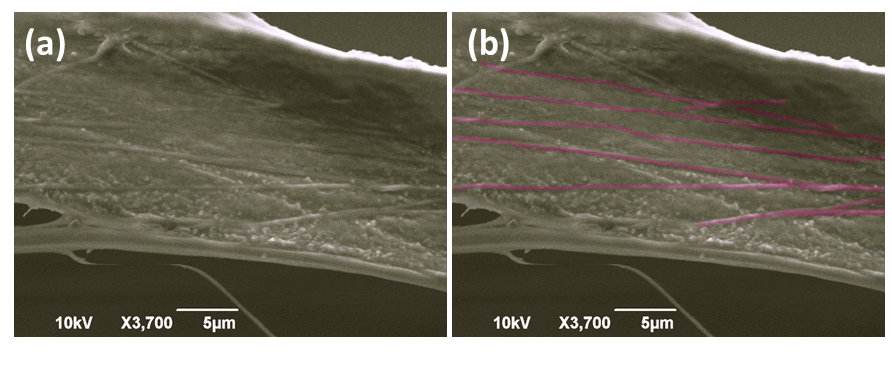


**Fig. S2** (a) 30° tilt view SEM of cell growing on suspended PVA-gelatin nanofiber (b) false-color showing nanofibers.


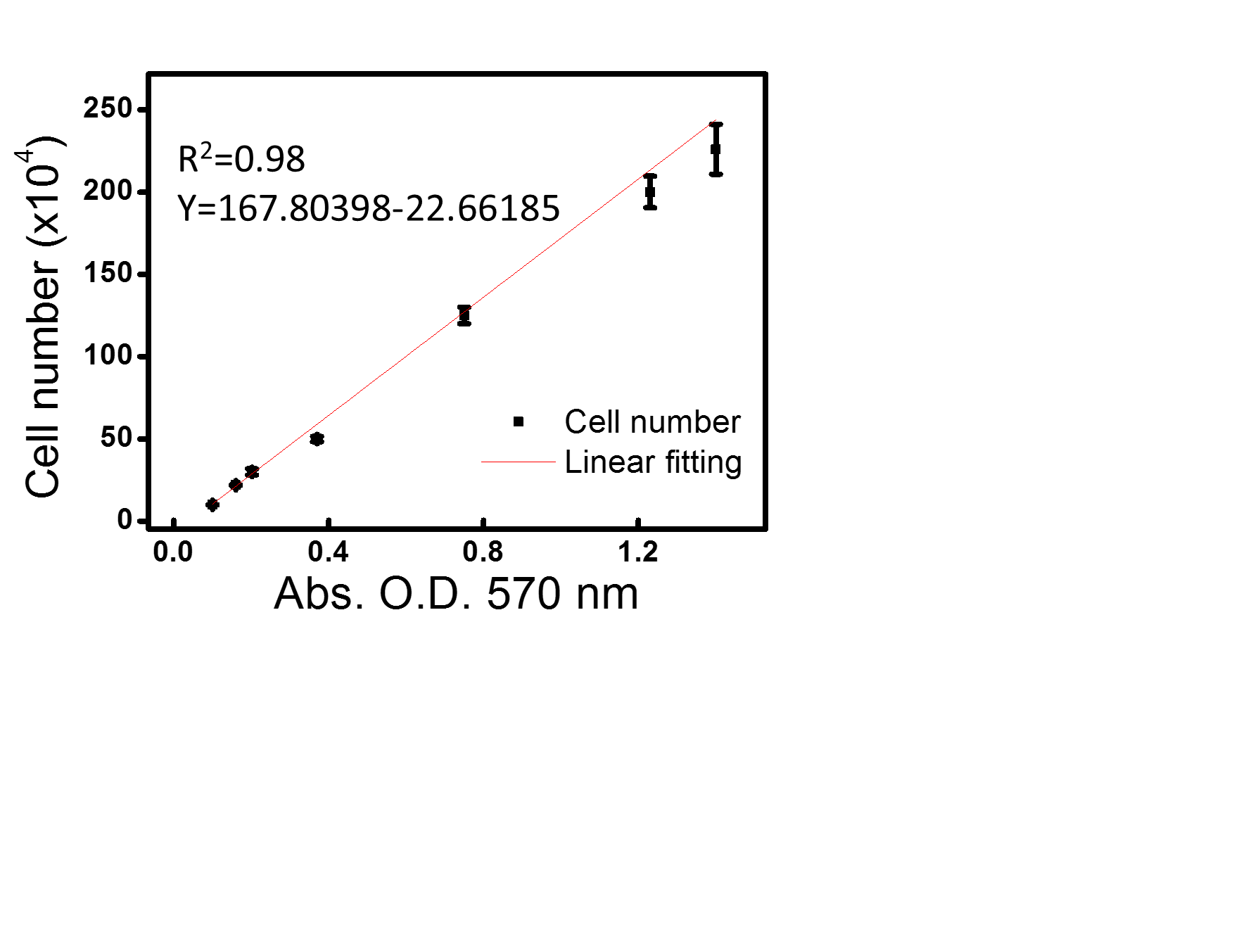


**Fig. S3** Absorbance of against number of cells
